# Review of “Analysis and application of analog electronic circuits to biomedical instrumentation” by Robert B Northrop

**DOI:** 10.1186/1475-925X-11-29

**Published:** 2012-06-15

**Authors:** Dobromir Dobrev

**Affiliations:** 1 FACET (Fabless Centre for Engineering & Test), 1138, Sofia, Bulgaria

## 

Book details:

Robert B. Northrop:

*Analysis and application of analog electronic circuits to biomedical instrumentation* Second edition, Biomedical engineering series, 2012, CRC Press, Taylor & Francis Group, 6000 Broken Sound Parkway NW, Suite 300, Boca Raton, FL 33487-2742, ISBN-10: 1439866694, ISBN-13: 978-1439866696, 578 pages, 15 chapters, Price: $119.95

The basic task of the Biomedical engineering consists of finding the adequate solutions for processing and analyzing the different types of biological signals. The nature of the real world is analog. The most of the body signals are also analog. During acquisition, they are frequently mixed with noise; that is why each biosignal acquisition system should start with well designed analog signal processing front-end. The practice of such design is as much art as science. Biomedical engineers have to combine technical knowledge of different disciplines with a great deal of theoretical background, experience and intuition, but they specifically must know the state of the art in the analog biomedical techniques. As a starting point, they need good books where to teach some secrets of development and implementation of up-to-date circuits, modules and devices in the field.

The second edition of ‘*Analysis and application of analog electronic circuits to biomedical instrumentation*’ helps biomedical engineers to understand the basic analog electronic circuits used for body signal acquisition. Since many bioelectric signals are within the microvolt range, the noise from electrodes, amplifiers, and the environment is often significant compared to the level of the useful signals. The book is intended for use in a classroom course on analog electronic circuits in Biomedical engineering for junior or senior undergraduate students. It also can serve as a reference book for biophysics and medical students interested in the topics. The book is organized into 15 chapters. It includes also an index, glossary, bibliography and recommended reading sections. Tasks for additional training, based on the discussed matter, are given at the end of some chapters. It should be mentioned the lack of hints or ready answers for solving them. Besides, it will be better for the reader, if the important formulas are highlighted in bold.

The material is well structured. It starts with the introduction of some mandatory basic states in this interdisciplinary field. The sources of bioelectric signals in nerves and muscles are discussed. The electrical properties of popular biomedical signals such as EMG and ECG are given together with characteristic of EMG and ECG amplifiers. Other biosignals such as EEG, EOG, ERG and EcoG are also described. The background ends with the very important electrical properties of bioelectrodes. Here I would like to add that for a completed work, an equivalent scheme of the body-electrodes-amplifier interface should be included together with discussion how the common mode (mainly power-line) interference is transformed into differential input voltage due to the electrode impedance imbalance in two- and three-electrode biosignal amplification techniques. The difference in electrode polarization voltages also is one of the main limitation factors to the amplifier first stage design.

Models for hand calculation of semiconductor devices used in analog electronic systems are further described. Junction and Schottky diodes, BJT, JFET and MOSFET devices, as well as the frequency behavior of the basic one and two transistor amplifiers are discussed. Photodiodes, LEDs and laser diodes are also presented. When I was going to read this chapter, I was giving thought instinctively to the nowadays available Spice models (Shockley for diodes, Ebers-Moll and Gummel-Poon for BJTs, Level 1 through BSIM3v3 for MOSFETs), and Spice based programs for modeling and simulation of analog circuits (PSpice, LTSpice, TINA Spice, XSpice, HSpice, Spice 3, Ngspice, Spice Opus, etc.). PSpice is the most popular program and serves as an industry standard for simulation of analog circuits with discrete components. Talking to device models, it would be very usefully to mentioned some PSpice models for diode, BJT, MOSFET (only the simple Level 1 model since the advanced BSIM3v3 and its later versions have a lot of parameters), and their model parameters. Some important device characteristics can be also shown as a result of dedicated circuit simulation schemes.

Next, the architecture of the differential amplifier is analyzed. Frequency behavior, differential mode and common mode gain, as well as CMRR are discussed. Special attention is given to differential amplifiers built with op amps. The important for medical applications schemes of two-op-amps and three-op-amps instrumentation amplifier, called in-amp, are described. The in-amp is a differential amplifier, which has very high input impedance and very high CMRR. Strictly speaking, the CMRR is the most important parameter of each differential amplifier since if it is 1 (or 0dB), the device amplifies equally differential and common mode signals. In such case it is not a differential amplifier and can be replaced by two single-ended amplifiers. In the two or three op amp in-amp circuits, the low-frequency CMRR depends mainly on mismatch of the used resistors. My note to this chapter 3 is related to the in-amp CMRR. It would be nice if a formula for the CMRR dependence on the tolerance of the used resistors was given. I appreciate the subchapter ‘How signal source impedance affects the low-frequency CMRR’, but will note that the signal source impedance in biosignal amplification can be disregarded compared to the electrode impedances; so it would be better if they were discussed in more details.

Attention is paid to the positive and negative voltage and current feedbacks, as well as to their affect on the amplifier performance. Amplifier feedback frequency response, the stability assessment by Bode plots, and the root-locus technique are presented. The usage of positive feedback in oscillator design is demonstrated for phase-shift and Wien bridge oscillators. Talking to stability, I find a discussion on the present day methods for stability evaluation would be very useful. Nowadays, there are two popular methods for such evaluation and for optimization of analog feedback system, and both of them are based on dedicated Spice simulations: loop gain evaluation in frequency domain (AC analysis) and pulse response evaluation in time domain (transient analysis). The first method consists of artificial breaking (blocking) the feedback loop for AC signals by inserting LC low-pass filter with artificially large time constant (in the range of teraseconds) into the loop, usually close to the amplifier input, and of injecting AC source at that point. The loop gain is evaluated for a phase margin during the AC analysis. The system is stable if the phase lag is below 115 degree and the loop gain equals 0 dB (corresponding to >65 degree phase margin). However, this is true only if the circuit is a pure minimum-phase system, where a given frequency response corresponds to well known phase response. If this is not the case, there is no correspondence between frequency and phase responses. In other words, there are two or more separate signal paths to the output and the feedback loop can not be properly broken, thus the phase margin result is not relevant. That is why, a second method is used too and the closed loop system is tested for its reaction to the input and output applied voltage or current pulses at transient analysis. The pulse response must not have oscillations or ringing.

The properties of op amps and its application in active filters are discussed in two consecutive chapters. It is shown how ideal op amp is interpreted in quick pencil-and-paper circuit analysis. Various practical op amps are described such as low-noise op amps, electrometer op amps, chopper stabilized op amps, power op amps, wide band and high slew rate op amps. Gain-bandwidth relations for voltage and current feedback op amps are given. Analog voltage comparators are introduced with practical circuit examples. Op amp based integrators and differentiators, charge amplifiers, and linear optocoupler isolation amplifiers are explained with their application in biomedical instrumentation. Sallen-Key active filters and filters based on three and four op amp biquads are described. Active filters with generalized impedance converters (GIC) are also in the scope of the chapter. Special attention is given to tunable active filters using analog multipliers and digital potentiometers. I note here that it would be better if a simple Spice first and second order op amp macro models were similarly discussed. I also mark that in most active filter simulations, the op amps can be modeled by a simple gain blocks (voltage controlled voltage source with gain 100k). Besides, applying dynamic offset cancellation techniques in op amp design (the so called zero-drift amplifiers), we can obtain excellent DC performance: DC gain, CMRR and PSRR beyond 120dB. The low-frequency noise (1/f) is canceled and the op amps manifest the thermal noise floor only. An example is OPA333 produced by TI, which has 1.1uVpp noise (0.01Hz – 10Hz), operates from 1.8V to 5.5V and consumes only 17uA.

Instrumentation amplifiers and medical isolation amplifiers are again discussed from another point of view. Integrated in-amp IC AD620 and three op-amp in-amp structures are considered. Isolation amplifiers with transformer coupling, opto coupling, capacitive coupling, and magneto-resistive coupling are discussed. The relatively new AD *icoupler* ADuM series of digital isolators with integrated chip-scale transformer are also mentioned. Since all amplifiers, used to record biopotential signals, must meet safety standards for breakdown voltage and maximum leakage current, a special attention was given to available safety standards for bioamplifiers and medical-grade power supplies. Here I will make the following comments. The in-amp AD620 is an ancient bipolar technology IC put on the market before 20 years. This IC is specified for ±18V operation and consumption of 1.3mA current. The tendency in the analog design is low-voltage and low-power operations. Low-voltage analog design in the term of modern CMOS technologies means a circuit operation with supply voltage below the sum of the two (nmos and pmos) threshold voltages (V_T_) and the two saturation voltages (V_dsat_). For example, let V_TN_ = V_TP_ = 0.6V and V_dsatn_ = V_dsatp_ = 0.2V; then the circuit design is low-voltage if it is able to operate with supply voltage below 1.6V! Of course, in medical electronics 1.6V is very low, and the low-voltage operation is considered to be with 3.3V or even 5V supply. I would like to mention TI in-amp IC INA322 (based on two op amp in-amp structure), which is designed for medical applications, operates from 2.7 to 5.5V, and consumes 40uA supply current. The INA322 gain is internally set to 5 (due to electrode polarization and common mode interference, this is the usual first stage gain coefficient), and can be increased by an external resistor if needed. One remark about the isolation: if low-power ADCs and microcontrollers are used, the popular front-end concept is to isolate the bioamplifier together with ADC or even low-power microcontroller, and then to use digital isolators and serial link to the non-isolated part.

The author considers in detail the noise and the design of low noise circuits. Starting with the probability density function and the autocorrelation function, the noise sources in BJTs, JFETs and amplifiers are discussed. It is shown how the noise is modeled in active devices by current and voltage noise sources. Propagation of noise through filters and equivalent noise bandwidth, spot noise and noise in differential amplifiers, as well as the noise factor and figure are considered. Cascaded noisy amplifiers and the effect of the feedback on the amplifier noise are also discussed. It is shown that for a low-noise system, only the first stage should be low-noise with a gain about 5 to 10. I appreciate also the subchapter dealing with lock-in amplifiers. Using synchronous detection, the lock-in amplifier becomes a powerful analog tool for precise measurements of frequency components, present in a given narrow frequency band. It can be mentioned that the lock-in amplifier performs analog ‘Fourier transform’ at one point, because its output is amplitude and phase of the frequency component at that point. I will note that although many examples of amplifier noise calculations are given, attention is paid only to the high frequency noise (thermal, Johnson or white noise). There are no examples of how low-frequency noise (1/f, flicker or pink noise) can be integrated and its rms value calculated from a given spectral density. Here I have another remark that capacitors also have a thermal noise, and what is more, they determine the value of thermal noise floor in the whole circuit. It is stated in page 288 that if feedback resistors are replaced by capacitors, they will not introduce thermal noise. This is truth but to some extent only. Let us consider simple RC first order low-pass filter. The standard deviation of resistor thermal noise (presented by its rms value) is e_n_ = sqrt(4kTRΔf). The equivalent noise bandwidth of a first order low-pass filter is Δf = (π/2)/(2πRC) = 1/(4RC). Replacing Δf in e_n_, R drops out, and e_n_ becomes e_n_ = sqrt(kT/C). The result shows that a noise floor in the circuit depends only on the capacitor value and temperature. So, the thermal noise on capacitors is referred as e_n_ = sqrt(kT/C*)*. Thus, 1pF capacitor has 64uVrms noise. The kT/C noise is a constraint in selecting the minimum capacitor value in switching capacitor circuits, where the SC noise does not depend on R_ON_ of the used switches. The noise is introduced rather by the thermodynamic equilibrium of the amount of charge on the capacitor than by the capacitor itself. Once the capacitor is disconnected from a circuit, the thermodynamic fluctuations are frozen at a random value, whose standard deviation is e_n_. The noise can be reduced by signal averaging that means a reduction of the frequency band. Since averaging by N, we reduce the noise by sqrt(N), the averaging e.g. by 4 will increase the resolution by 2 or 1bit.

Further, sampling and aliasing are discussed. DAC architectures with R-2R resistive matrix and binary weighted capacitive matrix, and split capacitor matrix for higher resolution (>8 bits) are considered. Various ADC architectures such as tracking ADCs, successive approximation ADCs, integrating ADCs, flash ADCs, sigma-delta ADCs are described. Static and dynamic characteristic of DACs and ADCs differential nonlinearity DNL and integral nonlinearity INL are presented. Sample and hold circuits, and quantization noise are discussed. Here it will be useful to mention the ECG/EEG front-end series of ICs from TI, which provides for a complete front-end for biopotential measurements. For example, IC ADS1298 integrates eighth 24-bit simultaneous sampling sigma-delta ADCs with input referred noise of 4uVpp and eighth PGAs with selectable gain from 1 to 12. The supply voltage range is 2.7-5.25V for the analog and 1.65-3.6V for the digital part. The IC has built-in oscillator, reference, DRL circuit, lead-off detection, pace detection, respiration impedance measurement and test signals generation. The data rate is up to 32ksps. The current consumption is 3mA for the analog part and 0.5mA for the digital part.

Other discussions are devoted to the modulation and demodulation techniques, as well as to the power amplifiers with their applications in biomedicine. The amplitude and frequency modulation are considered with the methods of their generation and demodulation. Delta modulation is also discussed. The authors pay heed to PLL circuits and their applications. BJT and MOSFET power amplifiers with transformer or complementary output stages are also examined. Special attention is given to class D amplifiers. Apex (now Cirrus Logic) power op amp series, efficiency and distortions in power op amps, and heat-sinking are discussed too. My comment is that the application of power amplifiers in electrical muscle stimulation and in electocoagulation for burning various skin lesions, warts, vessels, etc. deserves to be discussed too.

Wireless patient monitoring (WPM) and radiofrequency identification (RFID) tags are also considered. Wi-Fi, Zigbee and Bluetooth protocols, UHF transmitters and antennas are discussed together with revealing how WPM reduces the probability of patient microshock. RFID tags, GPS tags, ultrasonic tags and tag readers are examined. I appreciate the addendum of these chapters to the second edition of the book because the future of BME is coupled with wireless patient monitoring and RFID tags.

Finally, special analog circuits and systems used in biomedical instrumentation are discussed. Phase locked loop circuits, voltage and current controlled oscillators, phase sensitive rectifiers, analog multipliers, digital phase level and edge sensitive detectors, true RMS converters, and temperature sensors are examined. Three examples of medical instrumentation systems are given. I appreciate the presentation of the instrument for impedance plethysmography because the bioimpedance measurement is a classic method in the respiration monitoring, and therefore deserves to be discussed in more details. I have also the following comments. First, the standard 12 ECG leads and especially how they can be derived from the 8 independent body positions might be explained. Next, the so called ‘Driven Right Leg’ (DRL) circuit deserves to be discussed in details, since versions of DRL are implemented in almost all electrocardiographs. The DRL purpose is to drive the body through the right leg (reference) electrode by the opposite voltage in order to reduce the common mode voltage component at all amplifier inputs. So, the DRL circuit is a circuit with a common mode shunt-shunt negative feedback (parallel to the input voltage feedback), which actively reduces the impedance of the right leg electrode. The DRL circuit should have as high as possible loop gain, especially for the low-frequencies, i.e. for the 50Hz/60Hz power-line interference. The DRL stability is an interesting subject for Spice simulations. The DRL circuit simply is an opamp integrator stage. The human body usually has a stray capacitance to the earth in the range of 200pF and the present-day biosignal amplifiers are isolated. Therefore, the body-earth stray capacitance or the isolated ground capacitance represent major factor for the DRL instability, since they introduce an additional pole in the DRL control loop. Further on, the Automated External Defibrillators (AED) analyze the ECG usually acquired by the circuit used for shocking. AEDs have a high voltage part wherein a 47-100uF capacitor is charged up to 2000-4000V. During the shock, this capacitor is discharged through the chest impedance, very often by means of IGBT H-bridge to provide bidirectional current pulses (>10A). Holter monitors and pacemakers are other special circuits where the low-voltage and low-power design encounters major constrains. Medical imaging (ultrasound, magnetic resonance and computed tomography) might be also presented for basic information.

Summarizing the discussed topics of the book, I will emphasize the significance of a lot of analog electronic circuits and modules such as amplifiers, active filters, detectors, wireless transmitters, frequency and phase analyzers, etc. used for solving the specific problems of adequate acquisition, processing and analysis of biomedical information in real clinical conditions. Very often the signals are contaminated by different types of noise: power-line interference, drift, involuntary muscle disturbances, artefacts provoked by troubles in the electrode-to-skin contact, outside disorders and events discrediting the accuracy and the stability of data coming from sensors and transducers for conversion of non-electric parameters into electrical signals. The effective suppression and even elimination of the noise contributes to more precise diagnostic conclusions in the electrocardiography, electroencephalograpy, electromyography, electrooculography and other methods for data extraction from biomedical signals; monitoring in the intensive care; the medical imaging; the ultrasound examinations; during surgical interventions etc. Some special applications in the medical electronics, such as Holter type systems for long term recordings of vital patient data, wireless portable monitors, implantable pacemakers etc., put extremely high requirements for power efficiency and miniaturization that can be met only by means of modern CMOS processes and technologies moving into the less than 100nm nanoscale range. Therefore, the analog circuit design in the biomedical instrumentation will continue to be the background of the advance in biomedical research and the clinical medicine.

In conclusion, I would like to say that my comments to each chapter are based on what I had in mind when I was going through the pages. I should note that these comments are really minor remarks in regards to the whole material discussed in the book. Its content is very wide and reaches almost all topics of the analog design, implemented in the biomedical instrumentation. I can say that the book is bible in the field, and recommend it not only for students and biomedical engineers but also for experienced analog designers, who should have the book on their desks to use it as a reference in their every day job.

## Author information

Dr. Dobromir Dobrev has more than 15 years experience in BJT and CMOS analog and mixed-signal IC design (ASIC and ASSP), as well as in analog signal processing in Medical electronics.

## Competing interests

The author declares he has no competing interests.

